# Psychological resilience and anticipatory grief in the parents of children with cancer: The mediating role of pain catastrophizing and the moderating role of family resilience

**DOI:** 10.1016/j.apjon.2026.100999

**Published:** 2026-06-24

**Authors:** Di Zhang, Xiaofang Qiu, Jie Jiang, Funa Yang, Jiebing Luo, Hongtao Cheng, Zhengyang Hui, Caixia Wang, Kunhao Bai, Jun-e Zhang

**Affiliations:** a School of Nursing, Sun Yat−sen University, Guangzhou, China; b Cancer Prevention Center, Sun Yat−sen University Cancer Center, Guangzhou, China; c Nursing Department, Henan Cancer Hospital, Zhengzhou, China; d Department of Endoscopy, Sun Yat−sen University Cancer Center, Guangzhou, China

**Keywords:** Psychological resilience, Anticipatory grief, Pain catastrophizing, Family resilience

## Abstract

**Objective:**

To investigate the status of anticipatory grief, psychological resilience, family resilience, and parental pain catastrophizing about the child's pain in parents of children with cancer, and to identify factors associated with anticipatory grief. The study also explored the potential mediating role of parental pain catastrophizing about the child's pain in the relationship between psychological resilience and anticipatory grief, as well as the moderating role of family resilience.

**Methods:**

A total of 236 parents of children with cancer were recruited from four Grade A tertiary hospitals in China between November 2024 and April 2025. The participants were assessed using the Anticipatory Grief Scale (AGS), Connor–Davidson Resilience Scale (CD–RISC), Family Resilience Scale (FRS), and Pain Catastrophizing Scale (PCS).

**Results:**

The parents of pediatric cancer patients reported a mean AGS score of 76.74 (standard deviation [SD] = 16.92). Multiple linear regression showed that educational level, psychological resilience, family resilience, and pain catastrophizing were significantly associated with anticipatory grief, explaining 63.5% of the variance. Parental pain catastrophizing about the child's pain partially mediated the relationship between psychological resilience and anticipatory grief (indirect effect: *B* = −0.125, 95% confidence interval [CI]: [−0.182,−0.070]). Furthermore, family resilience moderated the pathway from psychological resilience to pain catastrophizing, with a significant psychological resilience × family resilience interaction (B = −0.319, P < 0.001). The index of moderated mediation was also significant (index = −0.061, 95% CI: [−0.102, −0.026]).

**Conclusions:**

Anticipatory grief in parents of children with cancer was directly associated with psychological resilience and indirectly associated with parental pain catastrophizing about the child's pain. Family resilience moderated the relationship between psychological resilience and pain catastrophizing, suggesting that higher family resilience may strengthen the protective association of psychological resilience against pain catastrophizing. These findings suggest that strengthening the protective function of family resilience may inform future supportive interventions and provide potential targets for clinical support aimed at alleviating anticipatory grief in parents and promoting overall family adjustment.

## Introduction

Approximately 429,000 new cases of childhood cancer are diagnosed globally each year.^1^ A review reported that the age-standardized global incidence rate of cancer among children is 141 cases per million per year.^2^ Childhood malignancies are typically highly aggressive, involving frequent pain episodes and a high risk of complications. These cancers frequently require prolonged treatment associated with substantial costs, with a marked negative impact on both the affected children and their parents.^3^ Parents, as the primary caregivers of these children, play a critical role in daily care, treatment decisions, and assisting with symptom management.^4^ However, the sustained stress associated with caregiving may progressively erode parental resilience. This not only may compromise the quality of care but also may exacerbate parental burnout, psychological distress, and anticipatory grief.^5^

Anticipatory grief is defined as a multifaceted emotional and cognitive process involving negative, distressing, and sorrowful reactions, accompanied by continual transformation and reconstruction of the individual's self−concept, triggered by the impending loss of someone or something of profound significance.^6^ Studies have shown that anticipatory grief may be associated with a range of physical and psychological symptoms, including sleep disturbances, appetite changes, fatigue, anxiety, emotional exhaustion, and social withdrawal, highlighting the need for ongoing emotional, psychological, cultural, and supportive care for affected family members.^7^^,^^8^ Without timely intervention, this can lead to a further decline in the family's ability to provide care. This decline in caregiving capacity intensifies fears of an unfavorable prognosis, thereby trapping parents in a vicious cycle of deepening emotional distress.^9^ However, the factors influencing anticipatory grief are complex. Existing research primarily focuses on the direct correlating factors of anticipatory grief,^10^ while the mechanisms underlying the interactions among these factors remain unclear.^11^ Therefore, a thorough investigation into the underlying psychological mechanisms that may be related to anticipatory grief in parents of children with cancer is crucial for providing theoretical guidance for targeted interventions, potentially benefiting both the children and their caregivers.^11^

Psychological resilience refers to the ability to maintain a positive mindset and psychological adaptability when confronted with major stressors, such as crises, suffering, adversity, and traumatic events.^12^ Existing research shows that the presence of psychological resilience is associated with faster recovery from emotional distress and may buffer against the negative effects of adverse emotions.^13^ It has also been found that psychological resilience may be associated with restored family function, may be related to higher family resilience, and may help maintain normal family functioning.^14^ Parents with higher levels of psychological resilience may be more likely to employ positive cognitive reappraisal and problem-focused coping strategies and may be better able to distinguish between “normal treatment responses” and “abnormal symptoms” during the child's treatment,^15^ which may be associated with less excessive worry and grief about pain and side effects.^16^ Evidence suggests that adequate levels of psychological resilience may counteract the detrimental associations of negative emotional states.^17^ However, existing studies have primarily highlighted the numerous benefits of psychological resilience and its potential value in coping with adverse crisis events.^18^ Nevertheless, the specific mechanisms by which psychological resilience relates to anticipatory grief in parents of children with cancer remain unclear,^11^ and the underlying pathways between these two factors have not been thoroughly elucidated. Additionally, there is a lack of exploration into potential confounding factors, leaving insufficient theoretical guidance for clinical interventions.^19^^,^^20^

Parental pain catastrophizing about the child's pain may be a key mediator in this mechanism. It is defined as an exaggerated negative cognitive-affective response to the child's actual or anticipated pain, characterized by rumination, magnification of the threat of the child's pain, and a perceived inability to cope.^21^ Children are typically limited in their ability to articulate pain, which may lead to a subjective amplification of the child's pain by the parents.^22^ Driven by the grief associated with the prospect of losing a child, parents may be prone to maladaptive cognitive assessments, automatically associating pain with negative outcomes, such as disease progression, poor prognosis, and even death.^23^ These maladaptive appraisals can intensify the fear and dread of potential loss, ultimately triggering or exacerbating anticipatory grief.^24^ Studies have shown that psychological resilience may act as an intrinsic protective factor and may buffer the impact of the child's pain on the caregiver, possibly by reducing parental pain catastrophizing about the child's pain, thereby potentially maintaining mental health and fostering positive emotional adaptation.^25^ Thus, it can be hypothesized that parental pain catastrophizing acts as a mediator in the relationship between psychological resilience and anticipatory grief. Current research has described the direct interactions and mutual associations between these variables, and has examined how other variables may relate to these pathways. However, the intrinsic interaction mechanisms among the three variables and their potential mediating pathways require further investigation to elucidate.

The resilience of the family provides crucial support during childhood cancer treatment, serving as a key bridge between the family and the individual.^26^ Family resilience denotes the capacity of a family to withstand adverse events and adjust positively by the effective leveraging of internal and external resources, supports, and strengths when confronting crises and risks.^27^ A further study indicated that family resilience can enhance psychological resilience, enable accurate cognitive transformation in parents by promoting continued positive communication, and provide emotional support and practical assistance,^28^ thereby potentially amplifying the beneficial associations of psychological resilience.^29^ Previous research suggests that the presence of even moderate psychological resilience in a caregiver can assist collaboration at the family level in reducing pain catastrophizing.^30^ Therefore, family resilience may strengthen the negative association between psychological resilience and pain catastrophizing, and further may be associated with the link between psychological resilience and anticipatory grief.

**Fig. 1:**
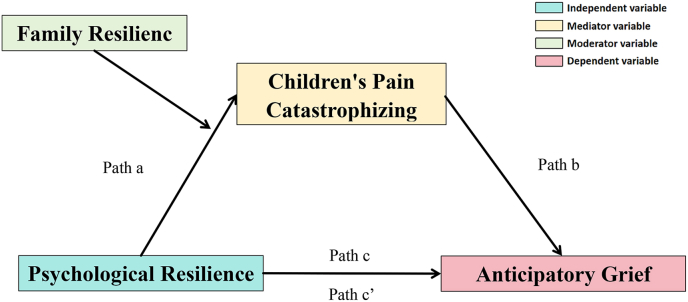
Hypothesized moderated mediation model.

Previous studies have primarily examined these variables in isolation without elucidating their combined mechanisms of action. This study aims to deepen our understanding of the mechanisms underlying anticipatory grief from both individual cognitive and family system perspectives. We seek to identify the potential mediating role of pain catastrophizing in the relationship between psychological resilience and anticipatory grief among parents of children with cancer, and to investigate whether family resilience may be associated with a stronger association between psychological resilience and pain catastrophizing. Based on Hill's ABC−X model of family stress,^31^ the present study examined mechanisms that may help explain anticipatory grief among caregivers of children with cancer. The cancer diagnosis of the child (A, the stressor) is hypothesized to be associated with the level of anticipatory grief in the caregiver (X, the crisis outcome) both directly and indirectly through the caregiver's catastrophizing of the child's pain (C, the cognitive appraisal). Additionally, psychological and family resilience (B, resources) are posited to buffer this relationship, directly associated with lower grief and indirectly associated through lower pain catastrophizing. Using the ABC−X model, this study aimed to (a) evaluate the current status of psychological resilience, family resilience, and anticipatory grief among parents of pediatric cancer patients, and (b) examine the moderating role of family resilience in the relationship between parental psychological resilience and catastrophizing the child's pain. An analytical framework was constructed based on these theoretical and empirical foundations (Fig. 1), leading to the following three hypotheses:

H1 Psychological resilience is negatively correlated with anticipatory grief.

H2 Parental pain catastrophizing about the child's pain partially mediates the relationship between the psychological resilience of the caregiver and their anticipatory grief.

H3 Family resilience moderates the association between the psychological resilience of the caregiver and the catastrophizing of the child's pain.

## Methods

### Sample source

This multicenter, cross-sectional study was conducted in the Hematology, Pediatrics, and Oncology Departments of four Grade III Class A hospitals in Zhengzhou, Wuhan, and Shenzhen, China. The parents of pediatric cancer patients were recruited using convenience sampling between November 2024 and April 2025.

### Participants

The required sample size was calculated using the multivariate correlation study sample size formula *N* = (U_α_ × S/*δ*)^2^.^32^ The significance level was set at *α* = 0.05, with U_α_ = 1.96, and a margin of error of *δ* = 0.2, with S representing the standard deviation. A preliminary survey was conducted with 30 parents of pediatric cancer patients. The standard deviations of the highest-scoring items from the scales measuring psychological resilience, anticipatory grief, family resilience, and pain catastrophizing scales were calculated, yielding scores of 3.47 ± 0.99, 2.95 ± 0.87, 3.15 ± 1.45, and 2.73 ± 0.89, respectively, and S = 1.45 (the maximum standard deviation observed in a pilot survey of 30 participants across the four key scales). The minimum number of valid responses required, calculated using this formula, was 201.92, which was rounded up to 202. Allowing for an anticipated invalid questionnaire rate of approximately 15%, at least 238 questionnaires were required to be distributed (202/0.85 = 237.65, rounded up to 238). In the formal survey, 250 questionnaires were distributed and returned. After excluding 14 invalid questionnaires, 236 valid responses were included in the final analysis, exceeding the minimum required number of valid responses.

#### Inclusion criteria

Caregivers who were parents of children meeting the following criteria were enrolled in the study: (1) The child was aged between 0 and 15 years; (2) The child had a histopathologically confirmed malignant tumor; (3) The child's diagnosis was made >1 month before enrollment (including newly diagnosed and relapsed cases). In addition, the caregiver was required to be: (1) Aged ≥18 years; (2) Fully informed of the child's diagnosis and have provided written informed consent to take part in the study; (3) Lacking in severe visual/hearing impairments, cognitive impairment, or a history of mental illness.

#### Exclusion criteria

Caregivers were excluded if either the child or the caregiver met any of the following conditions: (1) According to the evaluation and assessment by clinical medicine experts, the estimated survival period for the child is <6 months or the child is in a clinically critical/unstable condition; (2) The child did not live with the caregiver; (3) Caregiver history of severe trauma (e.g., domestic violence, sexual abuse, major accidental injury); (4) Caregiver having experienced other major traumatic events (e.g., bereavement, divorce) within the past month; (5) Presence of communication barriers in the caregiver that affect normal interaction, major illnesses, illiteracy, severe language/hearing impairment, or mental disorders.

### Measures

#### General information questionnaires

The questionnaire completed by the caregivers comprised 12 items and collected information on demographics, including gender, age, religious affiliation, marital status, occupation, educational level, average monthly household income, place of residence, current employment status, participation in health insurance, family history of cancer, and bereavement from the deaths of immediate family members (e.g., parents). The questionnaire for the children collected both demographic and disease information, including gender, age, diagnosis, current treatment phase, and treatment modalities.

#### Anticipatory Grief Scale (AGS)

The AGS was originally developed by Theut et al.,^33^ and was translated and adapted to Chinese culture by Zhou et al.^34^ The scale comprised 27 items with seven dimensions: anger (3 items), ability to complete tasks (3 items), guilt (4 items), irritability (4 items), anxiety (4 items), sadness (5 items), and sense of loss (4 items). Responses were recorded using a 5-point Likert scale ranging from 1 (“strongly disagree”) to 5 (“strongly agree”). The total score ranged from 27 to 135, with higher scores indicating more severe levels of anticipatory grief. The Cronbach's *α* of the AGS in this study was 0.92.

#### Family Resilience Assessment Scale (FRAS)

The FRAS was developed to evaluate family resilience by Chinese researchers.^35^ The scale consisted of 49 items with four dimensions: open communication (9 items), mutual support (11 items), family harmony (10 items), and perseverance (19 items). The responses were captured using a 5-point Likert scale, where 1 corresponded to “strongly disagree” and 5 to “strongly agree.” The total score ranged from 49 to 245, with higher scores indicating higher levels of family resilience. The Cronbach's *α* for the scale in this study was 0.96.

#### Connor–Davidson Resilience Scale (CD–RISC)

The CD–RISC was originally developed by Connor and Davidson (2003) and has been extensively validated and applied in various clinical settings.^36^ The Chinese version was compiled by Wu et al.^37^ The scale comprised 25 items with three dimensions: resilience (11 items), self-efficacy (8 items), and optimism (6 items). Responses were measured using a 5-point Likert scale, yielding a total score ranging from 0 to 100, with higher scores indicating greater psychological resilience. In the present study, the Cronbach's *α* for the CD−RISC was 0.94.

#### Pain Catastrophizing Scale (PCS)

This study used a parent-report version of the Pain Catastrophizing Scale (PCS), which was originally developed by Sullivan et al.,^38^ and subsequently adapted for parents to assess their catastrophizing thoughts about their child's pain.^39^^,^^40^ The scale consists of three dimensions: rumination (4 items), magnification (3 items), and helplessness (6 items), with a total of 13 items. Each item was rated on a 5-point Likert scale ranging from 0 (“never”) to 4 (“always”), with total scores ranging from 0 to 52. Higher scores indicate greater parental pain catastrophizing about the child's pain. In the present study, the Cronbach's *α* coefficient of the scale was 0.93.

### Data collection

Participants completed the questionnaire independently in a quiet room, with researchers providing standardized explanations as needed. A pilot study involving 30 participants (not included in the final sample) confirmed the feasibility of the protocol. In the formal study, participants were conveniently recruited from hematology and pediatric oncology wards, where research assistants proactively contacted and screened eligible subjects. After obtaining informed consent, participants were directed to an independent, quiet room. The questionnaires were distributed on-site in uniformly bound paper formats, and the survey was conducted via face-to-face, paper-and-pen self-administration. Researchers provided only necessary neutral explanations based on the standardized interpretation manual. The entire survey process took an average of approximately 24.3 minutes. Upon completion, research assistants immediately verified the completeness of each questionnaire and provided on-the-spot clarifications for any missing information or logical errors. All paper questionnaires were managed anonymously with unique numbering, subsequently entered independently by two individuals, and reviewed by a third party. Based on predefined invalidity criteria (e.g., predetermined response rates and patterned responses), 14 questionnaires were excluded from the 250 returned responses, resulting in 236 valid responses for analysis, yielding an effective response rate of 94.4%.

### Statistical analysis

Data were analyzed using SPSS 26.0 (IBM Corp., Armonk, NY, USA). Descriptive statistics were computed for all study variables. Categorical variables are summarized as frequencies (*n*) and percentages (%), while continuous variables are presented as means and standard deviations (SD). The Shapiro–Wilk test combined with the Q−Q plot was employed to assess the normality of continuous variables. The results demonstrated that the primary outcome indicators followed an approximately normal distribution. Accordingly, for indicators meeting the normality assumption and homogeneity of variance, independent samples *t*-tests or one-way ANOVA were used for intergroup comparisons; for indicators exhibiting non-normal distribution or heteroscedasticity, the Mann–Whitney U test or Kruskal–Wallis H test was applied. Bivariate Pearson correlation analysis was conducted to examine relationships between key variables. A moderated mediation analysis was performed using the PROCESS macro in SPSS.^41^ The analytical approach progressed from simpler to more complex models. PROCESS Model 4 was first used to examine the mediating role of pain catastrophizing in the relationship between caregiver psychological resilience and anticipatory grief. The significance of the indirect effect was evaluated using 5000 bootstrap samples, with a significant mediation effect indicated when the 95% bias-corrected confidence interval did not include zero. After the simple mediation model was established, PROCESS Model 7 was applied to examine whether family resilience moderated the association between psychological resilience and pain catastrophizing. Simple slope analyses were further conducted to examine the conditional effects of psychological resilience on pain catastrophizing at the mean, 1 SD below the mean, and 1 SD above the mean of family resilience. Statistical significance was set at P < 0.05, or when the 95% confidence interval did not include zero. Psychological resilience and family resilience were entered into the models using their original mean-item scores. No mean-centering or z-standardization was applied, and all coefficients are reported in the original mean-item score units.

## Results

### Association between general demographic characteristics and anticipatory grief

Tables 1 and 2 present the general demographic characteristics of participants and their children, along with the *t*-test and one-way ANOVA results. A total of 236 parents of children with cancer were enrolled in the study. As shown in Table 1, 52.5% parents were mothers, and most parents were aged between 30 and 49 years (72.9%). In terms of the children, 92.8% were the only child in the family, 30.5% were newly diagnosed, 34.3% were in the treatment period, and 35.2% were in the relapse and treatment stage (Table 2). The results showed that the educational level and monthly household income of the caregivers were significantly associated with anticipatory grief in univariate analyses.

**Table 1:** Anticipatory grief differences among different parents of children with cancer (*N* = 236).

Variable		*n* (%)	Mean ± SD	*t/F*	*P* value
Sex	Male	112 (47.5)	77.04 ± 17.83	0.257	0.797
	Female	124 (52.5)	76.47 ± 16.13		
Age, years	20-29	32 (13.6)	75.63 ± 17.98	1.586	0.193
	30-39	82 (34.7)	77.21 ± 16.69		
	40-49	90 (38.1)	78.66 ± 16.83		
	≥ 50	32 (13.6)	71.25 ± 16.23		
Religious belief	None	199 (84.3)	76.62 ± 17.27	−0.250	0.802
	Buddhism/Christianity/Islam	37 (15.7)	77.38 ± 15.10		
Marital status	Married	212 (89.8)	76.29 ± 16.74	−1.214	0.226
	Divorced/Widowed	24 (10.2)	80.71 ± 18.38		
Education level	Primary School and Below	25 (10.6)	87.96 ± 14.32	8.335	< 0.001
	Junior High School	38 (16.1)	84.87 ± 15.05		
	Senior High School/Technical Secondary School	68 (28.8)	76.03 ± 16.81		
	Junior College	45 (19.1)	71.51 ± 12.54		
	Bachelor's Degree and Above	60 (25.4)	71.63 ± 18.25		
Work status	Yes	215 (91.1)	76.61 ± 16.99	−0.358	0.721
	No	21 (8.9)	78.00 ± 16.53		
Medical insurance	Yes	220 (93.2)	77.13 ± 17.00	1.315	0.190
	No	16 (6.8)	71.38 ± 15.27		
Income (CNY per person per month and equivalent in USD)	≤ 2000 CNY (278 USD)	18 (7.6)	86.39 ± 13.17	10.327	< 0.001
	2001-4000 CNY (278-556 USD)	31 (13.1)	89.48 ± 13.58		
	4001-8000 CNY (556-1111 USD)	70 (29.7)	77.66 ± 18.44		
	8001-10,000 CNY (1111-1389 USD)	84 (35.6)	71.17 ± 15.56		
	≥10,000 CNY (1389 USD)	33 (14.0)	71.73 ± 12.57		

SD, standard deviation.

**Table 2:** Parents’ anticipatory grief differences among different children with cancer (*N* = 236).

Variable		*n* (%)	Mean± SD	*t/F*	*P* value
Sex	Male	113 (47.9)	77.39 ± 16.80	0.567	0.572
	Female	123 (52.1)	76.14 ± 17.08		
Age, years	0-4	77 (32.6)	76.88 ± 16.94	0.472	0.624
	5-9	76 (32.2)	78.03 ± 16.34		
	10-15	83 (35.2)	75.42 ± 17.53		
Whether the child is an only child	Yes	219 (92.8)	76.51 ± 16.96	−0.735	0.463
	No	17 (7.2)	79.65 ± 16.63		
Current disease/treatment phase	Newly-diagnosed	72 (30.5)	76.92 ± 17.09	0.259	0.772
	Treatment period	81 (34.3)	75.70 ± 17.54		
	Relapse and treatment	83 (35.2)	77.59 ± 16.30		
Treatment type	Regular chemotherapy	54 (22.9)	76.28 ± 17.59	1.838	0.161
	Radiotherapy	54 (22.9)	80.54 ± 16.60		
	Surgical treatment	128 (54.2)	75.33 ± 16.66		

SD, standard deviation.

The mean psychological resilience score was 71.06 (SD = 17.96), the mean pain catastrophizing score was 37.97 (SD = 10.95), and the mean family resilience score was 142.19 (SD = 37.10).

All scales employed in this study demonstrated high internal consistency, with Cronbach's *α* values ranging from 0.92 to 0.96. Our theoretical framework (Family Stress ABC−X Model) focuses on the overarching constructs of psychological resilience, family resilience, pain catastrophizing, and anticipatory grief rather than their specific subcomponents; consequently, all primary regression and mediation analyses utilized mean-item scores for each scale. Descriptive statistics in the tables are presented as total scores for interpretability. For enhanced transparency, descriptive statistics for each scale dimension/subscale are presented in Supplementary Table S1.

### Factors associated with anticipatory grief

The results of the correlation analysis indicated that anticipatory grief was negatively correlated with family resilience (*r* = −0.234, *P* < 0.01) and psychological resilience (*r* = −0.753, *P* < 0.01), but positively correlated with pain catastrophizing (*r* = 0.611, *P* < 0.01). Family resilience was positively associated with psychological resilience (*r* = 0.259, *P* < 0.01) and negatively associated with pain catastrophizing (*r* = −0.502, *P* < 0.01). Additionally, psychological resilience was significantly negatively correlated with pain catastrophizing (*r* = −0.578, *P* < 0.01) (Supplementary Table S2).

Multiple linear regression showed that educational level (*P* = 0.030), psychological resilience (*P* < 0.001), family resilience (*P* = 0.043), and pain catastrophizing (*P* < 0.001) were independently associated with anticipatory grief, explaining 63.5% of the variance (Table 3). Although household income was significantly associated with anticipatory grief in univariate analysis (Table 1, *F* = 10.327, *P* < 0.001), it did not retain significance in the multivariable model after adjusting for other variables (*P* = 0.052).

**Table 3:** Multiple linear regression analysis of factors associated with anticipatory grief (*N* = 236).

Variable	*B*	*SE*	*β*	*t*	*P* value
Constant	3.510	0.265	–	13.235	< 0.001
Educational level	−0.035	0.016	−0.096	−2.190	0.030
Income (CNY)	−0.048	0.025	−0.085	−1.953	0.052
Family resilience	0.078	0.039	0.095	2.031	0.043
Psychological resilience	−0.472	0.045	−0.541	−10.518	< 0.001
Pain catastrophizing	0.228	0.041	0.306	5.609	< 0.001

All regression coefficients were estimated using mean−item scores for each scale. *R*^*2*^ = 0.635 indicates the model explains 63.5% of the variance in anticipatory grief. *F* = 80.188, *P* < 0.001 confirms the overall model is statistically significant.

It is noteworthy that in the multivariable model, the regression coefficient for family resilience was positive (*B* = 0.078, *P* = 0.043), whereas the zero–order correlation between family resilience and anticipatory grief was negative (*r* = −0.234, *P* < 0.01). This sign reversal is statistically consistent with a suppression pattern. Specifically, family resilience shares positive variance with psychological resilience (*r* = 0.259) and negative variance with pain catastrophizing (*r* = −0.502). After controlling for both psychological resilience and pain catastrophizing—the two variables most strongly associated with anticipatory grief, the remaining unique variance of family resilience shows a positive association with anticipatory grief. This positive coefficient should not be misinterpreted as evidence that family resilience directly exacerbates grief, nor should it be taken as an independent protective effect. Rather, the sign reversal suggests that the negative zero-order correlation between family resilience and anticipatory grief (*r* = −0.234) may largely be attributable to its shared variance with higher psychological resilience and lower pain catastrophizing. However, because this interpretation is derived from a suppression pattern in a multiple regression model rather than from a formal test of the proposed mediating pathways, it should be regarded as exploratory and hypothesis-generating. Furthermore, given the cross-sectional nature of our data, causal inferences about the direction of these associations cannot be drawn. These findings tentatively suggest that any benefits of family resilience in relation to anticipatory grief may operate indirectly—potentially through enhancing individual psychological resilience and reducing catastrophic cognitive evaluations—rather than through direct pathways bypassing these individual-level processes.

### Testing the mediation model

The results of the mediation analysis are shown in Table 4. Psychological resilience (X) showed a significant negative total association with anticipatory grief (Y) (Path c, *B* = −0.605, 95% CI [−0.685, −0.526]). With the parental pain catastrophizing about the child's pain as the mediator (M), Path a (*B* = −0.655, 95% CI [−0.790, −0.520]) and Path b (*B* = 0.191, 95% CI [0.119, 0.263]) were both significant, and the direct association between X and Y remained significant (Path c', *B* = −0.480, 95% CI [−0.569, −0.391]). Bootstrapping analysis confirmed a significant partial mediating effect of M (*B* = −0.125, 95% CI [−0.182, −0.070]), accounting for 20.68% of the total association. Therefore, parental pain catastrophizing about the child's pain partially mediated the relationship between psychological resilience and anticipatory grief.

**Table 4:** The results of the mediation model (*N* = 236).

Model hypotheses		Meaning	*B*	95% CI		*SE*	*z/t*	*P* value	
				LLCI	ULCI				
Psychological resilience→ pain catastrophizing→Anticipatory grief	a∗b	Indirect effect	−0.125	−0.182	−0.070	0.028	−4.398	<0.001	Partial mediation
Psychological resilience→Pain catastrophizing	a	X→M	−0.655	−0.790	−0.520	0.068	−9.579	<0.001	
Pain catastrophizing→ anticipatory grief	b	M→Y	0.191	0.119	0.263	0.037	5.220	<0.001	
Psychological resilience→ anticipatory grief	c'	Direct effect	−0.480	−0.569	−0.391	0.045	−10.661	<0.001	
Psychological resilience→ anticipatory grief	c	Total effect	−0.605	−0.685	−0.526	0.040	−15.049	<0.001	

All models were adjusted for educational level and monthly household income as covariates. Mediation analysis with 5000 bootstrapping resamples. Indirect effect significance was determined by bias−corrected 95% confidence intervals (CI) excluding zero.

### Testing the moderated mediation model

The moderated mediation model was tested using PROCESS Model 7 with 5000 bootstrap resamples, adjusting for educational level and monthly household income as covariates (Table 5). The model predicting pain catastrophizing was significant (*P* < 0.001, *R*^*2*^ = 0.513), and the interaction between psychological resilience and family resilience in predicting pain catastrophizing was significant (B = −0.319, *P* < 0.001), indicating that family resilience moderated the relationship between psychological resilience and pain catastrophizing. The model predicting anticipatory grief was also significant (*P* < 0.001, *R*^*2*^ = 0.629), with psychological resilience (B = −0.480, *P* < 0.001) and pain catastrophizing (B = 0.191, *P* < 0.001) both independently associated with anticipatory grief. The index of moderated mediation was −0.061 (*Boot SE* = 0.019, 95% CI [−0.102, −0.026]) (Table 6), confirming that the conditional indirect effect was significantly moderated by family resilience. Conditional indirect effects at different levels of family resilience were: at low family resilience (M−1SD), indirect effect = −0.054 (95% CI [−0.095, −0.021]); at mean family resilience, indirect effect = −0.100 (95% CI [−0.152, −0.057]); and at high family resilience (M + 1SD), indirect effect = −0.146 (95% CI [−0.219, −0.082]) (Table 7). These results support a moderated mediation mechanism, in which the indirect effect of psychological resilience on anticipatory grief through pain catastrophizing was strengthened by higher levels of family resilience.

**Table 5:** The results of the moderated mediation model (*N* = 236).

	Anticipatory grief				Pain catastrophizing			
	B	*SE*	*t*	*P*	B	*SE*	*t*	*P*
Constant	3.925	0.213	18.402	<0.001∗∗	3.011	0.631	4.772	< 0.001∗∗
Psychological resilience	−0.480	0.045	−10.661	<0.001∗∗	0.398	0.219	1.816	0.071
Family resilience	–	–	–	–	0.490	0.206	2.374	0.018∗
Psychological resilience ∗ family resilience	–	–	–	–	−0.319	0.070	−4.579	< 0.001∗∗
Educational level	−0.032	0.016	−1.975	0.049∗	0.022	0.025	0.873	0.384
Household income	−0.045	0.025	−1.811	0.071	−0.017	0.038	−0.456	0.649
Pain catastrophizing	0.191	0.037	5.220	< 0.001∗∗	–	–	–	–
*N*	236				236			
*R**^2^*	0.629				0.513			
Adjust *R*^*2*^	0.621				0.500			
*F*	*F* (4,231) = 97.881, *P* < 0.001				*F* (5,230) = 48.448, *P* < 0.001			

Note: All coefficients were estimated using original mean-item scores. No mean-centering or z-standardization was applied. Simple slopes and conditional indirect effects were calculated at the mean, 1 SD below the mean, and 1 SD above the mean of family resilience. ∗*P* < 0.05 ∗∗*P* < 0.01; All models were adjusted for educational level and monthly household income as covariates. All coefficients were estimated using mean−item scores. Moderated mediation analysis with 5000 bootstrapping resamples.

**Table 6:** Indicators of moderating mediating effects in the moderating mediation model (*N* = 236).

Moderator variable	Mediator variable	Index	Boot SE	Boot LLCI	Boot ULCI
Family resilience	Pain catastrophizing	−0.061	0.019	−0.102	−0.026

All models were adjusted for educational level and monthly household income as covariates. All coefficients were estimated using mean−item scores. Moderated mediation analysis with 5000 bootstrapping resamples.

**Table 7:** Results of the indirect effects calculated using the mediation model conditions (N = 236).

Mediator variable		Moderator value	Effect	Boot SE	Boot LLCI	Boot ULCI
Pain catastrophizing	M−1SD	2.141	−0.054	0.019	−0.095	−0.021
	M	2.898	−0.100	0.024	−0.152	−0.057
	M+1SD	3.655	−0.146	0.035	−0.219	−0.082

Note: All coefficients were estimated using original mean-item scores. No mean-centering or z-standardization was applied. Simple slopes and conditional indirect effects were calculated at the mean, 1 SD below the mean, and 1 SD above the mean of family resilience. SD, standard deviation; CI, confidence interval.

### Results of the simple slope test

**Fig. 2:**
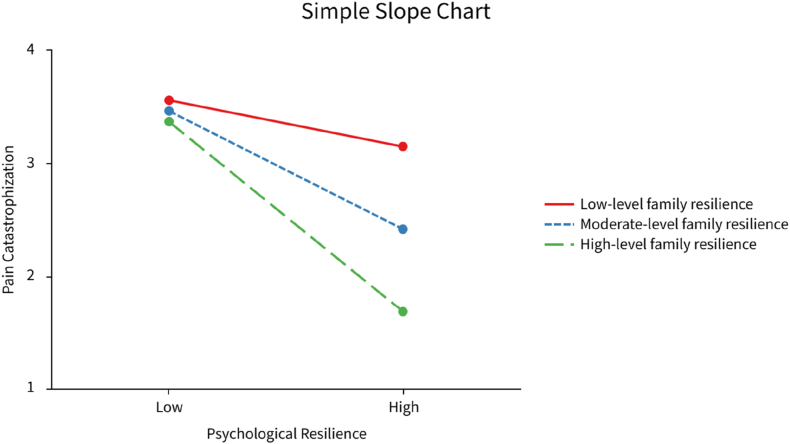
Simple slope plot illustrating the moderating effect of family resilience on the association between psychological resilience (mean-item score) and parental pain catastrophizing about the child's pain (mean-item score).

The simple slope analysis (Table 8) showed that the negative association between psychological resilience and pain catastrophizing was significant at low (*B* = −0.284, *P* = 0.001), mean (*B* = −0.525, *P* < 0.001), and high (*B* = −0.767, *P* < 0.001) levels of family resilience, with the association becoming stronger as family resilience increased. The hierarchical regression models examining the moderating effect of family resilience are presented in Supplementary Table S3, which reports the stepwise addition of the independent variable, moderator, and their interaction term, along with the corresponding model fit indices and variance inflation factors. Fig. 2 shows the simple slope plots illustrating the moderating effect across different levels of the moderating variable.

**Table 8:** The impact of Psychological Resilience on Pain Catastrophizing at different levels of Family Resilience (*N* = 236).

Level of family resilience	*B*	*SE*	*t*	*P* value	Boot LLCI	Boot ULCI
M	−0.525	0.061	−8.675	0.000	−0.645	−0.406
M+1SD	−0.767	0.074	−10.354	0.000	−0.912	−0.621
M−1SD	−0.284	0.086	−3.305	0.001	−0.454	−0.115

Note: All coefficients were estimated using original mean-item scores. No mean-centering or z-standardization was applied. Simple slopes and conditional indirect effects were calculated at the mean, 1 SD below the mean, and 1 SD above the mean of family resilience. SD, standard deviation; CI, confidence interval.

**Fig. 3:**
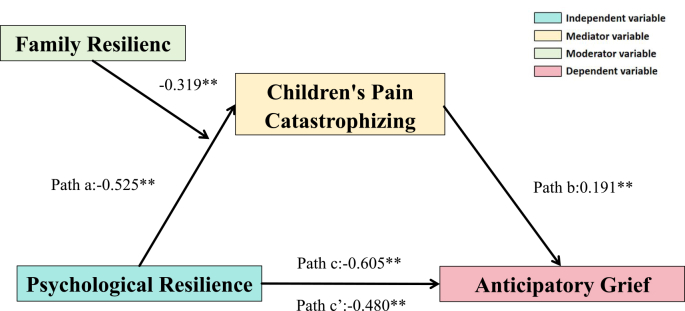
Final moderated mediation model with unstandardized path coefficients (mean-item score units). ∗∗*P* < 0.01.

Fig. 3 illustrates the pathway of the moderated mediation model. The path coefficients indicated that all relationships in the model were significant. After inclusion of the mediator (pain catastrophizing) and the moderator (family resilience), the direct effect of psychological resilience on anticipatory grief remained significant. Therefore, the link between psychological resilience and anticipatory grief was partly mediated by the parental pain catastrophizing about the child's pain, and the association between psychological resilience and pain catastrophizing was moderated by family resilience.

## Discussion

### Main findings

The study findings indicated that the parents of pediatric cancer patients experienced moderate levels of anticipatory grief. Anticipatory grief was found to be associated with educational level, household income, psychological resilience, family resilience, and pain catastrophizing. Interestingly, compared to the findings of a previous study on anticipatory grief levels in caregivers of adult cancer patients,^10^ the mean AGS score in the present study on caregivers of children (76.74 ± 16.92) was higher than that reported for caregivers of adults (69.41 ± 11.09). As has been suggested, unlike the caregivers of adult patients, who may be the children, spouses, or friends of the patient, the emotional bond parents have with their children is unique and intense, with the child often viewed as the “apple of their eye” (*“Zhang Shang Ming Zhu”* in Chinese). In addition, the traditional Chinese view of life, which interweaves the Confucian concept of destiny with folk beliefs in cause and effect, induces feelings of guilt in parents that sins committed in their earlier lives have brought consequences to the child, together with a sense of powerlessness of “Calling to the heavens yields no answer, calling to the earth yields no response” (*“Jiao Tian Tian Bu Ying, Jiao Di Di Bu Ling”* in Chinese), thereby may be associated with higher levels of anticipatory grief.

This study found that anticipatory grief was associated with both demographic and psychological factors, suggesting the importance of early identification of at-risk populations by health care professionals, followed by timely implementation of targeted psychological intervention strategies. Lower educational levels may limit parents' access to and understanding of medical information related to pediatric cancer, leading to difficulties in accurate interpretation of the diagnosis, treatment progress, and prognosis of their children's disease, thereby may be related to increased sense of uncertainty and fear about the disease.^42^ At the same time, a limited educational background may often restrict the social resources and occupational development space of parents, potentially reducing their social networks and social support. This may further weaken their ability to regulate emotions, potentially leading to the accumulation and intensification of negative emotions, such as anxiety, helplessness, and grief.^43^ Lower household income levels may also be associated with increased economic and caregiving burdens of parents.^8^ Economic constraints may force parents to make difficult choices in their children's treatment options, which may lead to self-blame and feelings of guilt. In addition, economic pressure may also be associated with reduced quality of life of the family, increased frequency of family conflicts, and further exacerbated feelings of helplessness and despair in the face of their children's illness, ultimately may be associated with a higher level of anticipatory grief.^9^

This study suggests that parental pain catastrophizing about the child's pain functions as a significant mediator in the relationship between psychological resilience and anticipatory grief. This finding differs from previous studies that solely focused on the direct associations of psychological resilience, instead highlighting the potential role of cognitive processing. Specifically, while most studies have reported that psychological resilience can negatively predict anticipatory grief,^44^^,^^45^ they failed to explain “how resilience exerts its influence.” Our study introduced pain catastrophizing, which may help clarify the pathway of “resilience → cognitive evaluation → grief.” Consistent with this conclusion, Lau et al. (2021) found that parents' accurate perception of their child's pain alleviated concerns about prognosis.^12^ Our study further suggests that such accurate perception may be viewed, to some extent, as a reflection of low catastrophizing, which in turn may be related to psychological resilience.^46^ Essentially, pain catastrophizing involves a subjective exaggeration of pain's meaning;^39^ together, these two processes constitute key components of cognitive processing, thereby potentially explaining the mechanism by which resilience operates at a more fundamental level.

Specifically, psychological resilience was associated with anticipatory grief through the mediating role of pain catastrophizing. Parents with lower levels of psychological resilience tended to exhibit more severe parental pain catastrophizing about the child's pain, which in turn was associated with higher anticipatory grief. This finding is consistent with the pain catastrophizing model proposed by Sullivan et al.,^47^ which posits that individuals' catastrophic interpretation of pain is associated with increased emotional distress and avoidance behaviors. The key distinction lies in the fact that Sullivan's model primarily draws from chronic pain patients themselves, whereas our study suggests that parents 'catastrophizing of their children's pain also elicits similar emotional consequences.^48^^,^^49^ Additionally, research on adult spouses with chronic pain has shown that observer catastrophizing of pain directly is associated with the spouse's depressive levels, without necessarily reducing their resilience.^50^ This discrepancy may stem from the more overt and stable pain expression observed in adult patients, whereas in children with cancer, pain responses are often concealed or distorted due to age-related limitations and fear, leading parents to overinterpret and remain highly vigilant.^51^ Consequently, in situations where pain signals are ambiguous and life-threatening, parents with lower psychological resilience may expend substantial cognitive resources on catastrophizing processes,^52^ thereby potentially amplifying the mediating effect in this sample. This may also help explain the pronounced tendency toward pain catastrophizing observed in our study population.^39^^,^^53^

It is noteworthy that the parents in this study were relatively sensitive to pain responses and exhibited high levels of pain catastrophizing. When faced with even minor changes in the child's treatment, the parents tended to overinterpret and become overly vigilant.^54^ Furthermore, pain catastrophizing, as a key mediating variable, was associated with the level of anticipatory grief and mediated the association between psychological resilience and anticipatory grief. Parents with higher levels of psychological resilience may internalize self-cognition, acquire relevant information, and correctly interpret pain responses in the child, which may help reduce levels of pain catastrophizing.^47^ Accurate perception of their children's pain may alleviate parents' concerns, enabling reductions in their associated fear of cancer, anxiety about prognosis, and levels of anticipatory grief.^12^ Therefore, confronting severe pain, adverse reactions to treatment, and potential complications in their children may induce fear and lead to pain catastrophizing in parents. This may further intensify their worries about the children's condition and future, ultimately may be associated with higher levels of anticipatory grief, thereby potentially forming a vicious cycle.^9^ Unlike previous arguments that attributed the vicious cycle solely to pain severity, our research suggests the association between pain catastrophizing and anticipatory grief, which may operate not only at the individual level but may also be reinforced or attenuated through family interactions.^55^ While some studies characterize pain catastrophizing as a relatively stable cognitive bias,^56^ our modeling results indicate that the catastrophic effect may be influenced by environmental factors—a finding that diverges from earlier theories. The potential explanation for this discrepancy lies in the recurrent and life-threatening nature of pain during cancer treatment, which maintains cognitive activation and renders individuals more susceptible to immediate modulation by family emotional resources.^57^ Future longitudinal studies across different disease entities and pain phases may further elucidate the state-trait structure of catastrophizing and the temporal dynamics of family resilience moderation.

A particularly noteworthy finding from the moderated mediation analysis was that family resilience moderates the relationship between parental psychological resilience and pain catastrophizing. High levels of family resilience may enable the rapid mobilization of both internal and external resources by the family when faced with the crisis of a child's cancer. Through positive and effective communication, mutual support, and collaboration, family members may adopt more positive coping strategies and rational responses to pain, which may help reduce the level of pain catastrophizing.^58^ This finding is highly consistent with the process-oriented model of family resilience, which posits that families achieve adaptive adjustments through effective communication, maintaining a positive attitude, and collaborative problem-solving when facing adversity.^59^ The present study identified a moderating role of family resilience on the first half of the pathway “psychological resilience → pain catastrophizing” rather than direct associations or a general moderation of the entire mediating chain. This implies that the protective role of family resilience may not directly be associated with lower anticipatory grief, but rather may operate by inhibiting caregivers' catastrophizing of their children's pain. Even with limited psychological resources, when the family system promptly provides emotional validation, information correction, and alternative interpretations, individuals are less likely to fall into the cognitive trap of pain catastrophizing.^46^^,^^60^^,^^61^ In the context of traditional Chinese culture, family resilience is deeply rooted in the values of harmony within the family, which leads to prosperity in all undertakings (*“Jia He Wan Shi Xing”* in Chinese). When faced with the crisis of a child's cancer diagnosis, families with high levels of resilience can rely on the traditional family style of rowing together through storms (*“Feng Yu Tong Zhou”* in Chinese), mobilize internal and external resources, promote the development of correct understanding and interpretation, and further reduce the degree of pain catastrophizing. If the caregiver within the family has low levels of psychological resilience, family members may blame one another, thereby further exacerbating feelings of helplessness and anticipatory grief.^13^^,^^54^ This study suggests a deeper underlying mechanism: mutual blame may be essentially an ineffective process of shared cognitive processing. Instead of correcting individuals' pain catastrophizing thoughts, it reinforces the erroneous consensus that “a child's pain indicates a worsening situation” through social confirmation effects, leading to collective amplification of catastrophizing within families. This mechanism explains why dysfunctional families exhibit more severe emotional consequences in response to pain compared to individuals engaging in catastrophizing.^57^ The suppression pattern observed in the multivariable regression further suggests that family resilience may exert its protective role indirectly.^29^ Clinically, this implies that interventions targeting family resilience should be delivered in conjunction with strategies to enhance individual psychological resilience and reduce pain catastrophizing, rather than as a standalone approach.

### Implications for nursing practice and research

Based on the findings of this cross-sectional study, several implications for clinical practice may be considered, though these suggestions require further validation in longitudinal or intervention studies before implementation. First, routine screening of parents' psychological resilience during diagnosis and key treatment stages using standardized scales could be incorporated into clinical workflows. Parents with low scores may be prioritized for psychosocial support.^62^ Second, oncology nurses may be encouraged to assess parents' maladaptive perceptions of their child's pain and to consider implementing nurse-led cognitive behavioral interventions and pain education programs aimed at correcting catastrophic interpretations such as “pain equals deterioration,” although the effectiveness of such programs should be evaluated in future research. Third, family-centered structured communication programs may be considered to support family resilience, with families exhibiting low resilience potentially referred to family therapy or peer support services, pending evidence from controlled trials. Fourth, the provision of accessible health education materials and financial assistance resources could help improve parents' understanding of the disease and reduce practical stressors.^6^ Finally, a tiered referral pathway could be considered, wherein parents with mild to moderate anticipatory grief might receive nurse-led psychological education and family-based support, while those with severe or persistent symptoms may be referred to clinical psychology or psychiatry for specialized care. The ultimate goal of these recommendations would be to reduce anticipatory grief and strengthen family adaptive functioning^6^;^63^ however, direct evidence for the efficacy of these approaches in reducing anticipatory grief remains limited and requires further investigation.

### Limitations

This study has several limitations. First, a convenience sampling method was employed to recruit participants from four tertiary hospitals located in major cities (Zhengzhou, Wuhan, and Shenzhen). This sampling strategy may limit the generalizability of the findings to rural areas, community hospitals, or parents from diverse cultural backgrounds. Second, the cross-sectional design cannot determine causal relationships or directional associations between psychological resilience, pain catastrophizing, family resilience, and anticipatory grief. Although we tested a moderated mediation model based on theoretical assumptions (the ABC−X model), the temporal sequence of these variables could not be established from cross-sectional data; longitudinal studies are required to validate the proposed causal pathways. Third, the examined models did not incorporate direct clinical measures of pediatric pain severity or disease severity, making it impossible to assess the extent to which observed correlations were influenced by actual clinical conditions. Fourth, all data were collected via self-report questionnaires, introducing potential common method bias as well as recall or social expectation biases; significant correlations among variables may have been exaggerated due to shared measurement variance. Fifth, we excluded children with an estimated survival <6 months. This may limit the generalizability of our findings to parents of children with the poorest prognoses, who may experience the highest levels of anticipatory grief and should be included in future studies. Sixth, reliance solely on quantitative self-report scales limits interpretative depth; qualitative methods could provide richer insights into parents' firsthand experiences of anticipatory grief; future studies should consider mixed-method designs to explore deeper underlying mechanisms.

## Conclusions

The findings suggest that family resilience moderates the association between psychological resilience and pain catastrophizing, thereby indirectly influencing anticipatory grief in parents of children with cancer. These results underscore the importance of close monitoring of family dynamics, promoting parental psychological well-being, and actively mobilizing internal and external family resources to support adaptive cognitive appraisals. In clinical practice, we recommend screening parents for psychological resilience and pain catastrophizing, implementing nurse-led cognitive interventions and pain education, enhancing family communication, establishing a phased referral system to potentially alleviate caregivers' anticipatory grief and improve the quality of child care.

## CRediT authorship contribution statement

**Di Zhang:** Conceptualization, Methodology, Writing − Original Draft. **Xiaofang Qiu:** Methodology, Writing. **Jie Jiang**: Data Curation, Visualization. **Funa Yang**: Resources, Supervision. **Jiebing Luo**: Supervision. **Hongtao Cheng**: Software, Validation. **Zhengyang Hui:** Project Administration. **Caixia Wang:** Formal Analysis. **Kunhao Bai:** Writing – Review & Editing. **Jun−e Zhang:** Writing – Review & Editing. All authors have read and approved the final manuscript.

## Ethics statement

This cross-sectional study was conducted in accordance with the Declaration of Helsinki (as revised in 2013). Ethical approval was obtained from the lead Ethics Committee of Henan Cancer Hospital (Approval No. 2024-279-002), and reliance approvals were obtained from all participating tertiary hospitals. Before data collection, all participants were provided with detailed information about the study purpose, procedures, voluntary participation, right to withdraw at any time without penalty, and data confidentiality. Written informed consent was obtained from all eligible parents of children with cancer prior to their inclusion in the study.

## Data availability statement

The datasets are not publicly available due to privacy and ethical restrictions but are available from the corresponding author on reasonable request.

## Declaration of generative AI and AI−assisted technologies in the writing process

During the preparation of this work the author(s) used DeepSeek and ChatGPT in order to refine language. After using this tool/service, the author(s) reviewed and edited the content as needed and take full responsibility for the content of the publication.

## Funding

This work was supported by the 10.13039/501100001809National Natural Science Foundation of China (Grant No. 82403973). The funder had no role in the study design, data collection, data analysis, manuscript preparation, or the decision to submit for publication.

## Declaration of competing interest

The authors declare no conflict of interest. The corresponding author, Prof. Jun−e Zhang, is an editorial board member of *Asia–Pacific Journal of Oncology Nursing*. The article was subject to the journal's standard procedures, with peer review handled independently of Prof. Zhang and the research groups.
