# Supplementary material for: Psychological resilience and anticipatory grief in the parents of children with cancer: The mediating role of pain catastrophizing and the moderating role of family resilience
Source: Asia Pac J Oncol Nurs. 2026 Jun 24;13:100999. doi: 10.1016/j.apjon.2026.100999 (PMC13380066; doi:10.1016/j.apjon.2026.100999)
Supplement: Multimedia component 1 [file mmc1.docx]

Supplementary Table S1 Descriptive statistics of subscales/dimensions for each instrument (*N* = 236)

| Instrument | Subscale/Dimension | Number of items | Mean | SD |
| --- | --- | --- | --- | --- |
| AGS (Anticipatory Grief Scale) | Anger | 3 | 8.59 | 2.50 |
|  | Ability to complete tasks | 3 | 8.39 | 2.43 |
|  | Guilt | 4 | 11.31 | 2.86 |
|  | Irritability | 4 | 11.23 | 2.90 |
|  | Anxiety | 4 | 11.38 | 3.22 |
|  | Sadness | 5 | 14.31 | 4.45 |
|  | Sense of loss | 4 | 11.54 | 3.19 |
| CD-RISC (Connor-Davidson Resilience Scale) | Resilience | 11 | 31.23 | 7.83 |
|  | self-improvement | 8 | 22.69 | 6.80 |
|  | Optimism | 6 | 17.14 | 4.67 |
| FRAS (Family Resilience Assessment Scale) | Open communication | 9 | 26.44 | 7.94 |
|  | Mutual support | 11 | 31.91 | 8.34 |
|  | Family harmony | 10 | 28.97 | 8.01 |
|  | Perseverance | 19 | 54.87 | 14.96 |
| PCS (Pain Catastrophizing Scale) | Rumination | 4 | 11.66 | 3.57 |
|  | Magnification | 3 | 8.80 | 2.83 |
|  | Helplessness | 6 | 17.52 | 5.38 |

Supplementary Table S2 Correlations among key variables (*N* = 236)

| Variables | 1 | 2 | 3 | 4 |
| --- | --- | --- | --- | --- |
| 1 Anticipatory Grief | 1.000 |  |  |  |
| 2 Family Resilience | -0.234** | 1.000 |  |  |
| 3 Psychological Resilience | -0.753** | 0.259** | 1.000 |  |
| 4 Pain Catastrophizing | 0.611** | -0.502** | -0.578** | 1.000 |

Note: **Represents a significance level of *P* < 0.01

Supplementary Table S3 Analysis Results on the Moderating Role of Family Resilience in the Pathway from Psychological Elasticity to Pain Catastrophization (*N* = 236)

| Model | term | Non-standardized coefficient | | β | *t* | *P* value | 95% CI | collinearity diagnostics | |
| --- | --- | --- | --- | --- | --- | --- | --- | --- | --- |
|  |  | B | SE |  |  |  |  | VIF | Tolerance |
| model 1 | Constant | 3.070 | 0.169 | - | 18.151 | 0.000^**^ | 2.737 ~ 3.403 | - | - |
|  | Educational Level | -0.001 | 0.029 | -0.001 | -0.020 | 0.984 | -0.057 ~ 0.056 | 1.193 | 0.838 |
|  | household income | -0.044 | 0.044 | -0.058 | -0.988 | 0.324 | -0.131 ~ 0.044 | 1.194 | 0.838 |
|  | Psychological resilience | -0.655 | 0.068 | -0.559 | -9.579 | 0.000^**^ | -0.790 ~ -0.520 | 1.189 | 0.841 |
|  | F | *F* (3,232)=39.265, *P*=0.000 | | | | | | | |
|  | *R*^2^；Adjust *R^2^* | *R^2^*=0.337, Adjust *R^2^*=0.328 | | | | | | | |
|  | *△R^2^*；*△F* | *△R^2^*=0.337, *△F* (3,232)=39.265, *P*=0.000 | | | | | | | |
| model 2 | Constant | 2.908 | 0.153 | - | 18.979 | 0.000^**^ | 2.606 ~ 3.210 | - | - |
|  | Educational Level | 0.018 | 0.026 | 0.036 | 0.690 | 0.491 | -0.033 ~ 0.069 | 1.204 | 0.831 |
|  | household income | -0.017 | 0.040 | -0.023 | -0.435 | 0.664 | -0.096 ~ 0.061 | 1.203 | 0.831 |
|  | Psychological resilience | -0.567 | 0.062 | -0.484 | -9.088 | 0.000^**^ | -0.690 ~ -0.444 | 1.232 | 0.812 |
|  | Family Resilience | -0.423 | 0.056 | -0.380 | -7.569 | 0.000^**^ | -0.533 ~ -0.313 | 1.096 | 0.913 |
|  | F | *F*(4,231)=50.916, *P*=0.000 | | | | | | | |
|  | *R*^2^；Adjust *R^2^* | *R^2^*=0.469, Adjust *R^2^*=0.459 | | | | | | | |
|  | *△R^2^*；*△F* | *△R^2^*=0.132, *△F*(1,231)=57.289, *P*=0.000 | | | | | | | |
| model 3 | Constant | 2.938 | 0.147 | - | 19.966 | 0.000^**^ | 2.648 ~ 3.228 | - | - |
|  | Educational Level | 0.022 | 0.025 | 0.044 | 0.873 | 0.384 | -0.027 ~ 0.071 | 1.205 | 0.830 |
|  | household income | -0.017 | 0.038 | -0.023 | -0.456 | 0.649 | -0.093 ~ 0.058 | 1.203 | 0.831 |
|  | Psychological resilience | -0.525 | 0.061 | -0.448 | -8.675 | 0.000^**^ | -0.645 ~ -0.406 | 1.260 | 0.794 |
|  | Family Resilience | -0.415 | 0.054 | -0.373 | -7.742 | 0.000^**^ | -0.521 ~ -0.309 | 1.097 | 0.912 |
|  | Psychological Resilience * Family Resilience | -0.319 | 0.070 | -0.215 | -4.579 | 0.000^**^ | -0.456 ~ -0.181 | 1.038 | 0.963 |
|  | F | *F*(5,230)=48.448, *P*=0.000 | | | | | | | |
|  | *R*^2^；Adjust *R^2^* | *R^2^*=0.513, Adjust *R^2^*=0.502 | | | | | | | |
|  | *△R^2^*；*△F* | *△R^2^*=0.044, *△F*(1,231)=20.970, *P*=0.000 | | | | | | | |

Note: * p<0.05 ** p<0.01; Dependent Variable = Pain Catastrophization
